# Development of a Porcine Model of Pulmonary Ischemia–Reperfusion Injury Relevant to Post-Esophagectomy Acute Respiratory Distress Syndrome

**DOI:** 10.3390/medsci14040490

**Published:** 2026-08-18

**Authors:** Mohammadreza Hafezi, Arash Saffari, Elias Khajeh, Christa Flechtenmacher, Christoph Lichtenstern, Arianeb Mehrabi, Camelia Garoussi

**Affiliations:** 1 Department of General, Visceral and Transplantation Surgery, Heidelberg University, Im Neuenheimer Feld 420, 69120 Heidelberg, Germany; mohammadreza.hafezi@med.uni-heidelberg.de (M.H.); elias.khajeh@med.uni-heidelberg.de (E.K.); arianeb.mehrabi@med.uni-heidelberg.de (A.M.); 2 Department of Pathology, Heidelberg University, Im Neuenheimer Feld 224, 69120 Heidelberg, Germany; 3 Department of Anesthesiology, Heidelberg University, Im Neuenheimer Feld 420, 69120 Heidelberg, Germany

**Keywords:** acute respiratory distress syndrome (ARDS), ischemia–reperfusion injury, esophagectomy, swine model, animal model, inflammatory cytokines

## Abstract

**Background**: Acute respiratory distress syndrome (ARDS) occurs in 20–40% of patients following esophagectomy and is associated with substantial postoperative morbidity and mortality. A major contributor to postoperative ARDS is pulmonary ischemia–reperfusion injury (IRI); however, the role of IRI in ARDS following esophagectomy is not adequately addressed in current experimental models. In this study, we established a large animal model of pulmonary IRI that reproduces key physiological, inflammatory, and histopathological features of pulmonary ischemia–reperfusion-induced acute lung injury relevant to postoperative ARDS after esophagectomy. **Methods**: Sequential pulmonary ischemia–reperfusion injury was induced in ten anesthetized Landrace pigs using unilateral hilar inflow occlusion. Right lung ischemia was achieved by clamping the hilar inflow for three hours, followed by reperfusion. Subsequently, the left lung underwent two hours of ischemia. Hemodynamic, respiratory, and inflammatory parameters were continuously monitored throughout the experiment. Blood samples were collected to assess leukocyte counts and circulating inflammatory cytokines, including tumor necrosis factor-α and interleukin-6. Lung tissue samples were obtained for histopathological evaluation. **Results**: ARDS-like lung injury was successfully induced in all animals, with PaO_2_/FiO_2_ ratios falling below 200 mmHg during the predefined reperfusion observation period. Lung compliance decreased by approximately 50% after ischemia and further declined following reperfusion. Progressive leukocyte elevation and elevated tumor necrosis factor-α and interleukin-6 levels were observed, indicating a systemic inflammatory response. Hallmark features of ARDS were histologically confirmed, including intra-alveolar hemorrhage, interstitial and perivascular edema, and neutrophil infiltration. **Conclusions**: This porcine model reproduces key physiological, inflammatory, and histopathological features consistent with pulmonary ischemia–reperfusion-induced ARDS-like lung injury. Although it does not reproduce the complete clinical syndrome of post-esophagectomy ARDS, it provides a clinically relevant translational platform for investigating pulmonary ischemia–reperfusion injury and evaluating potential preventive and therapeutic strategies.

## 1. Introduction

Esophageal cancer is one of the leading causes of cancer-related mortality worldwide, causing over 480,000 deaths annually [1,2,3]. The primary treatment for advanced esophageal cancer is esophagectomy, which is a complex and invasive procedure associated with high postoperative morbidity and mortality due to pulmonary complications [4,5]. These complications include acute respiratory distress syndrome (ARDS), which affects 20–40% of patients and contributes to 14% of mortality after surgery [5,6,7,8]. ARDS is also associated with severe morbidity following esophagectomy, characterized by alveolar and endothelial injury, inflammation, and impaired gas exchange, which can progress to multi-organ failure if untreated [9,10].

Pulmonary ischemia–reperfusion injury (IRI) is considered an important contributing mechanism to postoperative pulmonary complications, including ARDS, after esophagectomy [5,11,12]. During transthoracic phase of esophagectomy, right lung collapse and one-lung ventilation may cause transient pulmonary ischemia, followed by reperfusion after reconstruction, leading to IRI and subsequent pulmonary complications [13]. Although pulmonary IRI may contribute to the development of postoperative ARDS, it has not been well represented in experimental models. This means that, despite significant advancements in anesthetic and surgical techniques, postoperative IRI-induced ARDS remains a major challenge because we cannot properly elucidate the underlying mechanisms or evaluate therapeutic strategies [11,14,15]. Many existing experimental ARDS models often involve systemic inflammation, delayed injury onset, or limited clinical relevance to postoperative ARDS [5]. In short, current experimental models do not adequately replicate the perioperative ARDS pathophysiology seen in esophagectomy patients, which limits their utility in investigating the mechanisms of ARDS and testing therapeutic strategies.

In this study, we aimed to establish reproducible large-animal model of pulmonary IRI that reproduces key mechanisms in the development of ARDS-like lung injury relevant to the postoperative setting. The purpose of this model was to provide insights into the mechanisms of postoperative ARDS and to replicate key features such as endothelial and alveolar injury, inflammatory responses, and pulmonary vascular obstruction. Through histopathological analyses and biomarker identification, this model was developed as an experimental platform for investigating postoperative lung injury and evaluating potential therapeutic strategies.

## 2. Methods

### 2.1. Animals and Ethical Approval

All animal experiments were approved by the governmental authority of Karlsruhe (AZ 35-9185.81/G-85/12, 12 November 2012) and were conducted in accordance with the German Animal Welfare Act and institutional guidelines for the care and use of laboratory animals. The study was designed and reported in accordance with the ARRIVE 2.0 guidelines (Supplementary File). A total of 12 Landrace pigs (7 female, 5 male), aged 2–3 months and weighing 29.8 ± 3.4 kg, were enrolled in the study. Before initiation of the definitive study, the first two animals were prospectively designated as pilot experiments to assess the technical feasibility of the model and to optimize and standardize the surgical protocol. These pilot animals were not intended for inclusion in the final statistical analysis. Following successful protocol establishment, the remaining 10 animals constituted the definitive study cohort and all completed the experimental protocol according to the predefined study design. The final sample size of 10 animals was considered sufficient to achieve the objectives of this exploratory model-development study. In accordance with the principles of the 3Rs (Replacement, Reduction, and Refinement), no additional animals were included once the study objectives had been achieved. No animals were excluded because of intraoperative complications, protocol deviations, or mortality.

To minimize animal use, all experimental procedures and assessments were conducted sequentially in each animal following induction of ARDS. Animals were housed for 2–3 days before experimentation in species-appropriate facilities at the Interdisciplinary Biomedical Research Facility, Heidelberg University, with daily veterinary care. All experiments were performed in dedicated large-animal operating rooms.

### 2.2. Anesthesiological Management

Animals were fasted for 12 h with free access to water. Premedication consisted of intramuscular azaperone (4–8 mg/kg), midazolam (0.5–0.7 mg/kg), and ketamine (20 mg/kg). Anesthesia was induced intravenously via an ear vein using propofol (3–5 mg/kg). Following orotracheal intubation with a 7.5-mm tube (Rüsch™, Teleflex Medical GmbH, Fellbach, Germany), animals were mechanically ventilated (Primus, Dräger Medical, Lübeck, Germany). Continuous monitoring included capnometry and pulse oximetry.

Anesthesia was maintained using sevoflurane (minimum alveolar concentration 1 ± 0.2) in a gas mixture of oxygen (0.5–1.0 L/min) and room air (0.5–1.0 L/min), with blood gas and electrolyte monitoring. Neuromuscular blockade was achieved with pancuronium (3 mg bolus, repeated as required). Initial pressure-controlled ventilation settings were a respiratory rate of 15–20/min, a tidal volume of 7–8 mL/kg, an inspiratory:expiratory ratio of 1:2, a positive end-expiratory pressure (PEEP) of 5 cm H_2_O, and an FiO_2_ of 0.3. Ventilation was adjusted to maintain PaCO_2_ between 35 and 45 mmHg.

### 2.3. Instrumentation and Hemodynamic Monitoring

After induction of anesthesia, arterial and central venous access was established via bilateral cervical incisions along the medial border of the sternocleidomastoid muscle. On the right side, the internal and external jugular veins and the common carotid artery were exposed, cranially ligated, and caudally catheterized using an open cut down technique. On the left side, the external jugular vein was catheterized for infusion and hemodynamic management. All catheters (16G French) were connected to a pressure-monitoring system (Lohmeier AK 11).

Arterial blood samples were taken for blood gas analysis, and venous blood samples were taken for laboratory testing. Volume management consisted of an initial 500 mL saline bolus, followed by continuous infusion (200–300 mL/h) to maintain central venous pressure (CVP) of ≥6–7 mmHg. A suprapubic bladder catheter (Cystofix^®^, B. Braun, Melsungen, Germany) was placed to monitor urine output.

### 2.4. Physiological Monitoring

Mean arterial pressure (MAP), heart rate, and central vein pressure (CVP) were continuously recorded. Oxygen saturation was measured using pulse oximetry via a probe attached to the ear. Core body temperature was measured rectally.

### 2.5. Surgical Procedure and ARDS Model

Following intubation and instrumentation, transthoracic echocardiography was performed to exclude cardiogenic pulmonary edema and heart failure. ARDS was then induced to mimic the clinical scenario following esophagectomy. A right-sided anterolateral thoracotomy was performed through the fourth intercostal space. After opening the pleural cavity, the hila of both lungs were exposed, the perihilar pleura incised, and the hilar vessels prepared for clamping.

After completion of the surgical preparation, a 30-min stabilization period occurred (Phase 0). The hilar inflow of the right lung was then clamped for 3 h to induce ischemia (Phase 1). Subsequently, the right lung was declamped and reperfused (Phase 2), immediately followed by clamping of the left lung hilar inflow for 2 h (Phase 3). The subsequent 2-h observation period was divided into four predefined 30-min intervals (Phases 3a–3d):

- **Phase 0**: Stabilization period (30 min) before right hilar inflow clamping.
- **Phase 1:** Clamping of the right lung’s hilar inflow for 3 h.
- **Phase 2**: Immediate reperfusion of the right lung.
- **Phase 3**: Clamping of the left lung’s inflow immediately after reperfusion of the right lung, maintained until the end of the experiment (2 h) in four consecutive investigation phases in 30-min intervals:
  ○ ***Phase 3a***: 30 min after reperfusion.
  ○ ***Phase 3b***: 60 min after reperfusion.
  ○ ***Phase 3c***: 90 min after reperfusion.
  ○ ***Phase 3d***: 120 min after reperfusion.

Following 2 h of left hilar clamping, the animals were euthanized without reperfusion of the left lung. This approach was intentionally chosen to preserve the established severe impairment in pulmonary gas exchange, as reperfusion of the left lung would have partially restored pulmonary function and potentially obscured the physiological manifestation of ARDS-like lung injury at the final assessment.

**Figure 1:**
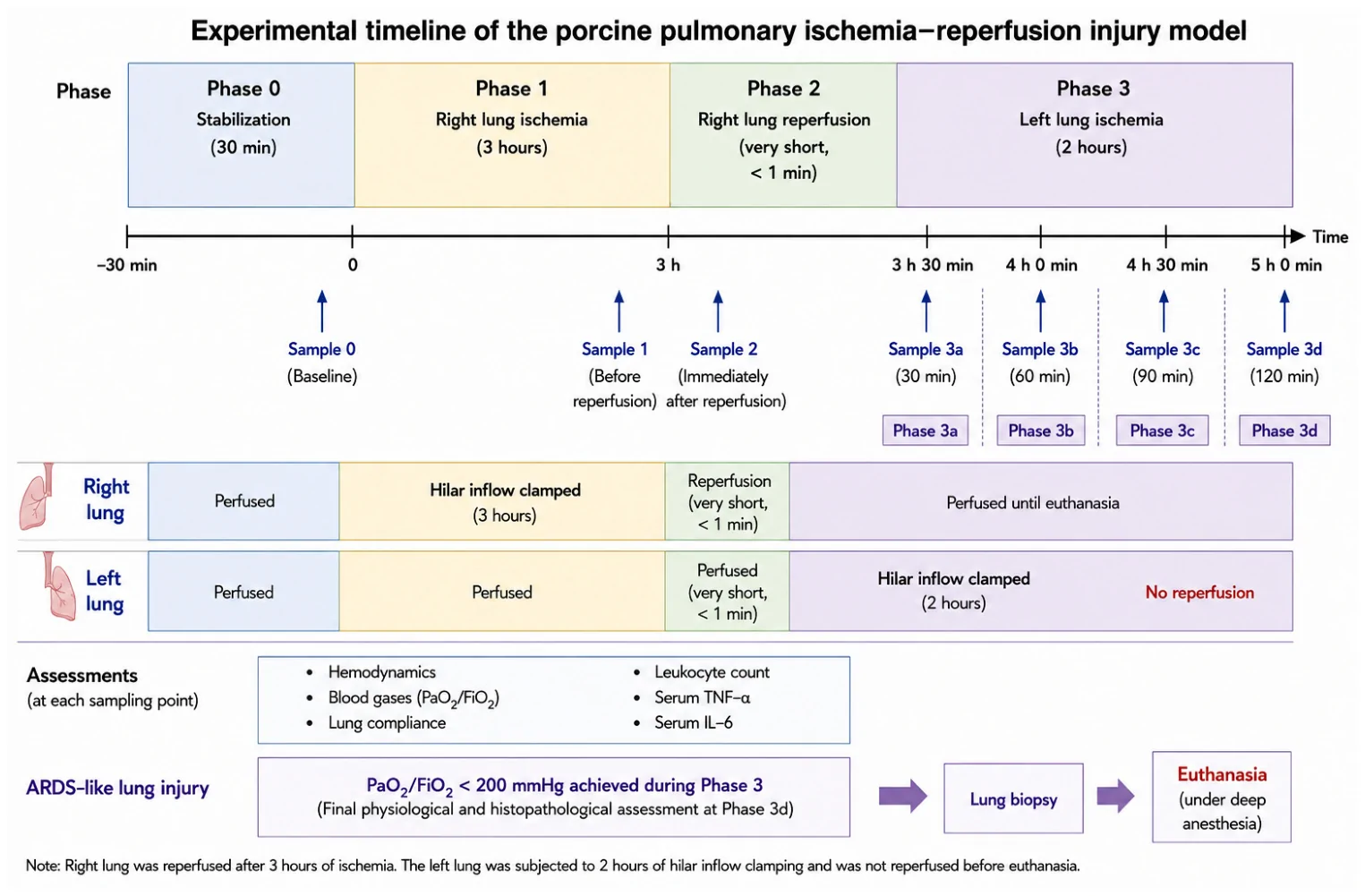
Schematic overview of the experimental protocol. Experimental timeline of the porcine pulmonary ischemia–reperfusion injury model showing the stabilization period (Phase 0), right lung ischemia (Phase 1), right lung reperfusion (Phase 2), left lung hilar clamping without reperfusion (Phase 3), predefined sampling time points, physiological assessments, and euthanasia.

At the end of the experiment, animals were euthanized under deep anesthesia by intravenous administration of potassium chloride (7.4%, 1 mL/kg). The complete experimental protocol is schematically illustrated in Figure 1.

### 2.6. Ventilatory Management During Ischemia

During lung ischemia, pressure-controlled ventilation was maintained with a PEEP of 5 cm H_2_O and tidal volume 6 mL/kg. The respiratory rate was adjusted to maintain a PaCO_2_ between 35 and 45 mmHg. FiO_2_ was initially set to 0.3 and adjusted to maintain adequate oxygen saturation. To minimize barotrauma, the ventilation mode and peak inspiratory pressure were reassessed and adjusted every 30 min during unilateral ischemia.

### 2.7. Definition of ARDS-like Lung Injury

ARDS-like lung injury was defined using predefined physiological and histopathological criteria. The primary physiological criterion was a PaO_2_/FiO_2_ ratio < 200 mmHg, supported by characteristic histopathological findings. These criteria were fulfilled during the predefined reperfusion observation period (Phase 3). As this was an experimental animal model, the complete clinical Berlin Definition was not applied [16].

### 2.8. Data Collection and Laboratory Analysis

At predefined phases, cardiovascular parameters (heart rate, MAP, CVP) and respiratory variables (pH, PaO_2_/FiO_2_ ratio, lung compliance) were recorded simultaneously. Venous blood samples were collected and aliquoted for leukocyte counts and measurement of the pro-inflammatory cytokines, tumor necrosis factor-α (TNF-α), and interleukin-6 (IL-6). Serum samples were analyzed immediately whenever possible. If immediate analysis was not feasible, samples were stored at 2–8 °C for up to 24 h and, for longer storage, frozen at −20 °C until analysis.

IL-6 concentrations were measured in serum using the Immulite 2000 IL-6 system (Siemens Healthineers, Erlangen, Germany), employing a sequential solid-phase chemiluminescent immunometric assay with a measuring range of 2–1000 pg/mL. Results were expressed as pg/mL. TNF-α concentrations were determined in serum by an accredited external reference laboratory (Labor Limbach, Heidelberg, Germany) using a chemiluminescent immunoassay (CLIA), Results were reported in ng/mL.

Serum samples were collected during the study phases as follows:

- **Sample 0 (Baseline)**: Before right hila clamping.
- **Sample 1**: Immediately before reperfusion of the right lung.
- **Sample 2**: Immediately after reperfusion of the right lung.
- **Sample 3**: During the clamping of the left lung in 30-min intervals.
  ○ ***Sample 3a***: 30 min after reperfusion.
  ○ ***Sample 3b***: 60 min after reperfusion.
  ○ ***Sample 3c***: 90 min after reperfusion.
  ○ ***Sample 3d***: 120 min after reperfusion.

### 2.9. Histopathology Analysis

Histopathological assessment was performed by a pathologist blinded to the experimental phase. Lung biopsies were obtained from the right lung before induction of ARDS and at the end of the experiment after the PaO_2_/FiO_2_ ratio had fallen below 200 mmHg. A total of 24 biopsy samples (1 cm^3^) were fixed in 4% formalin, processed routinely, embedded in paraffin, sectioned at 4 µm, and stained using the Leucognost^®^ NASDCL technique (Merck KGaA, Darmstadt, Germany).

Stained samples were assessed under the microscope at 40× magnification for intra-alveolar hemorrhage, perivascular edema, and interstitial edema. Specimens fulfilling all three predefined criteria were classified as ARDS-like lung injury. In addition, neutrophils were quantified in both intra-alveolar and interstitial/perivascular compartments in five randomly selected high-power fields (40×), and the mean count per specimen was calculated. A validated lung injury score or diffuse alveolar damage score was not applied.

### 2.10. Statistical Analysis

Data were analyzed using SPSS for Windows (Version 28.0, IBM Corp., Armonk, NY, USA). Continuous variables are presented as mean ± standard deviation. Temporal changes in cardiocirculatory, respiratory and inflammatory parameters were analyzed using repeated-measures analysis of variance (RM-ANOVA). Sphericity was assessed using Mauchly’s test, and Greenhous–Geisser correction was applied when the assumption of sphericity was violated. A two-sided *p*-value < 0.05 was considered statistically significant. Quantitative histopathological data (neutrophil counts) were compared between control and ARDS specimens using a paired Student’s *t*-test. A two-sided *p* value < 0.05 was considered statistically significant. No missing data occurred during the study; therefore, no imputation or special handling of missing values was required. As the primary objective of the statistical analysis was to evaluate overall temporal changes during the experimental protocol, no post-hoc pairwise comparisons were performed.

## 3. Results

The first two animals were predefined pilot experiments used to optimize and standardize the surgical and experimental protocol to ensure reproducibility of ARDS induction and were not included in final statistical analyses. The ARDS model was established and evaluated in the remaining 10 animals. All data are presented as mean values and were collected before, during, and after ARDS induction.

### 3.1. Cardiovascular Parameters (Heart Rate, MAP, CVP)

Cardiovascular variables during ARDS induction are summarized in Table 1. The heart rate was comparable during unilateral lung ischemia and immediately following reperfusion, with a delayed increase to a maximum elevation of 38% at 120 min after reperfusion (*p* = 0.001). MAP decreased significantly (*p* < 0.001) by approximately 30% from the baseline during ischemia and reperfusion, then increased 30 min after reperfusion. MAP increased by 27%, followed by normalization and a subsequent decline of 32% in the 120 min after reperfusion. CVP decreased significantly (*p* < 0.001) by 34% relative to baseline, indicating a redistribution of intravascular volume during ischemia–reperfusion. Aside from transient MAP reductions during lung clamping and early reperfusion, cardiovascular parameters remained stable throughout the protocol. Overall, the hemodynamic response was consistent and homogeneous across animals, confirming a controlled and reproducible experimental course.

### 3.2. Respiratory Parameters (pH, PaO_2_/FiO_2_ Ratio, Lung Compliance)

**Figure 2:**
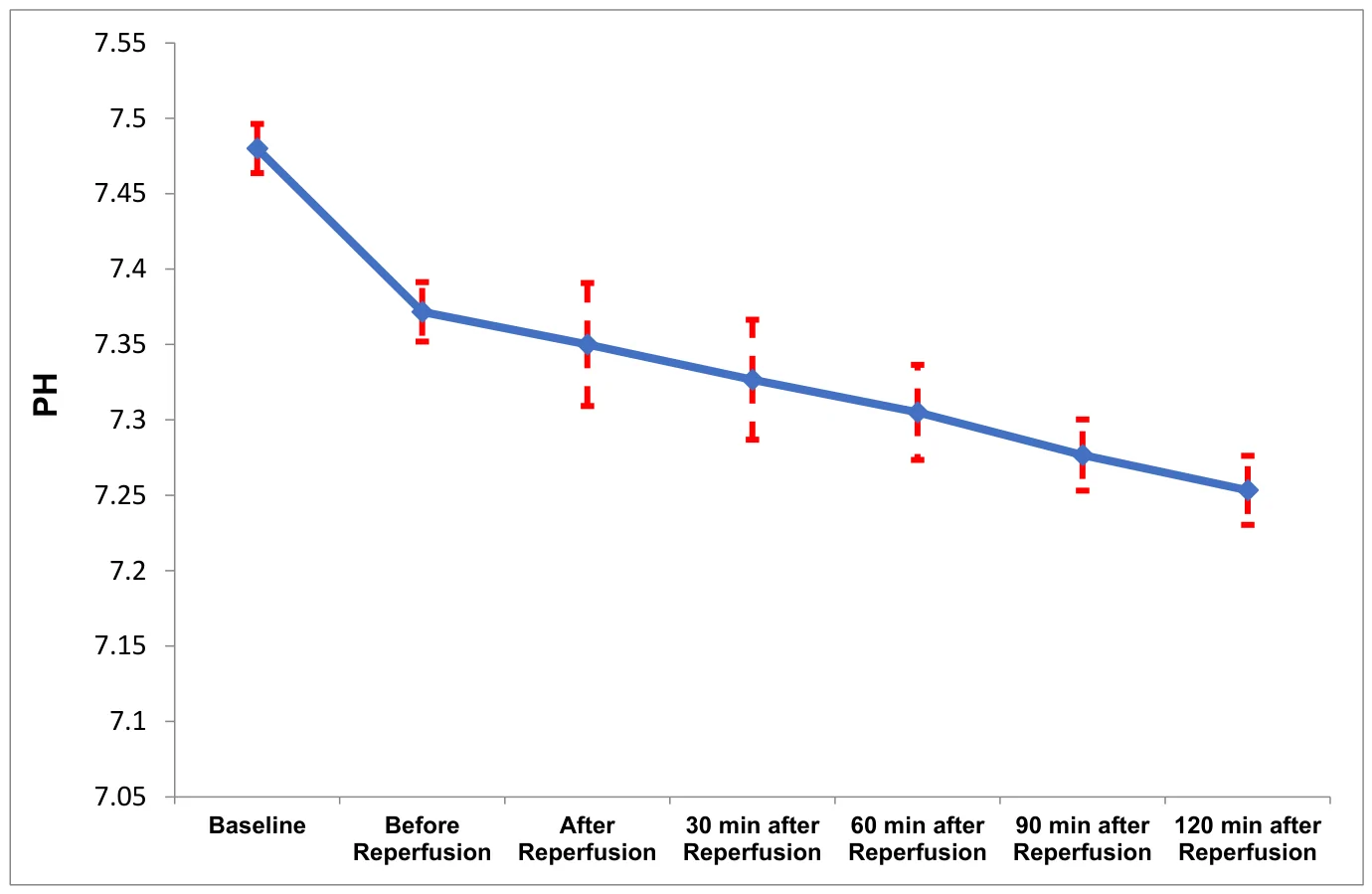
Changes in arterial pH in a porcine model of IRI-induced ARDS. *Baseline: sample 0; before reperfusion: sample 1; after reperfusion: sample 2; 30 min after reperfusion: sample 3a; 60 min after reperfusion: sample 3b; 90 min after reperfusion: sample 3c; 120 min after reperfusion: sample 3d*.

The arterial pH declined significantly after IRI-induced ARDS (*p* = 0.003), consistent with progressive metabolic acidosis. This decrease persisted throughout the ischemic phase and after reperfusion, reaching the lowest mean value of 7.24 at 120 min after reperfusion (Figure 2). Concurrent respiratory acidosis was effectively counteracted by ventilatory adjustments.

**Figure 3:**
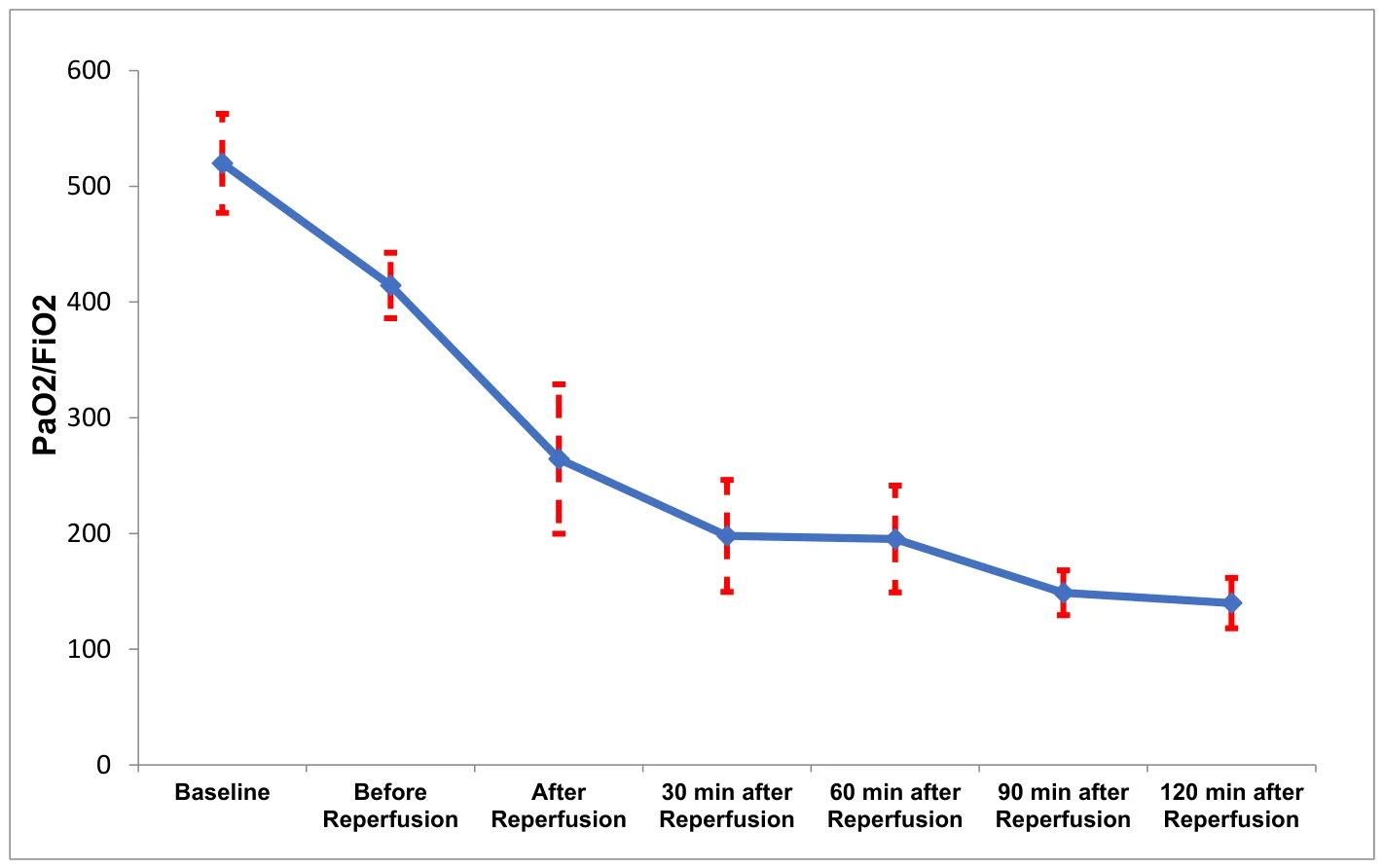
Changes in the PaO_2_/FiO_2_ ratio in a porcine model of IRI-induced ARDS. *Baseline: sample 0; before reperfusion: sample 1; after reperfusion: sample 2; 30 min after reperfusion: sample 3a; 60 min after reperfusion: sample 3b; 90 min after reperfusion: sample 3c; 120 min after reperfusion: sample 3d*.

Lung oxygenation was measured using PaO_2_/FiO_2_ ratios. At baseline, these ratios were homogeneous across all animals (Figure 3). During unilateral lung ischemia and throughout reperfusion, repeated measures ANOVA confirmed a highly significant temporal decline in PaO_2_/FiO_2_ ratios following ischemia–reperfusion injury (*p* < 0.001). At 30 min after reperfusion, the mean PaO_2_/FiO_2_ ratios fell below 200 mmHg and remained persistently low throughout the observation period. At the final assessment (120 min after reperfusion), the mean PaO_2_/FiO_2_ ratio had fallen to 139 mmHg. All animals met the predefined physiological oxygenation criterion corresponding to the moderate ARDS range, supported by histopathological evidence of acute lung injury. Final physiological and histopathological assessments were performed immediately before euthanasia following completion of the left hilar clamping period.

**Figure 4:**
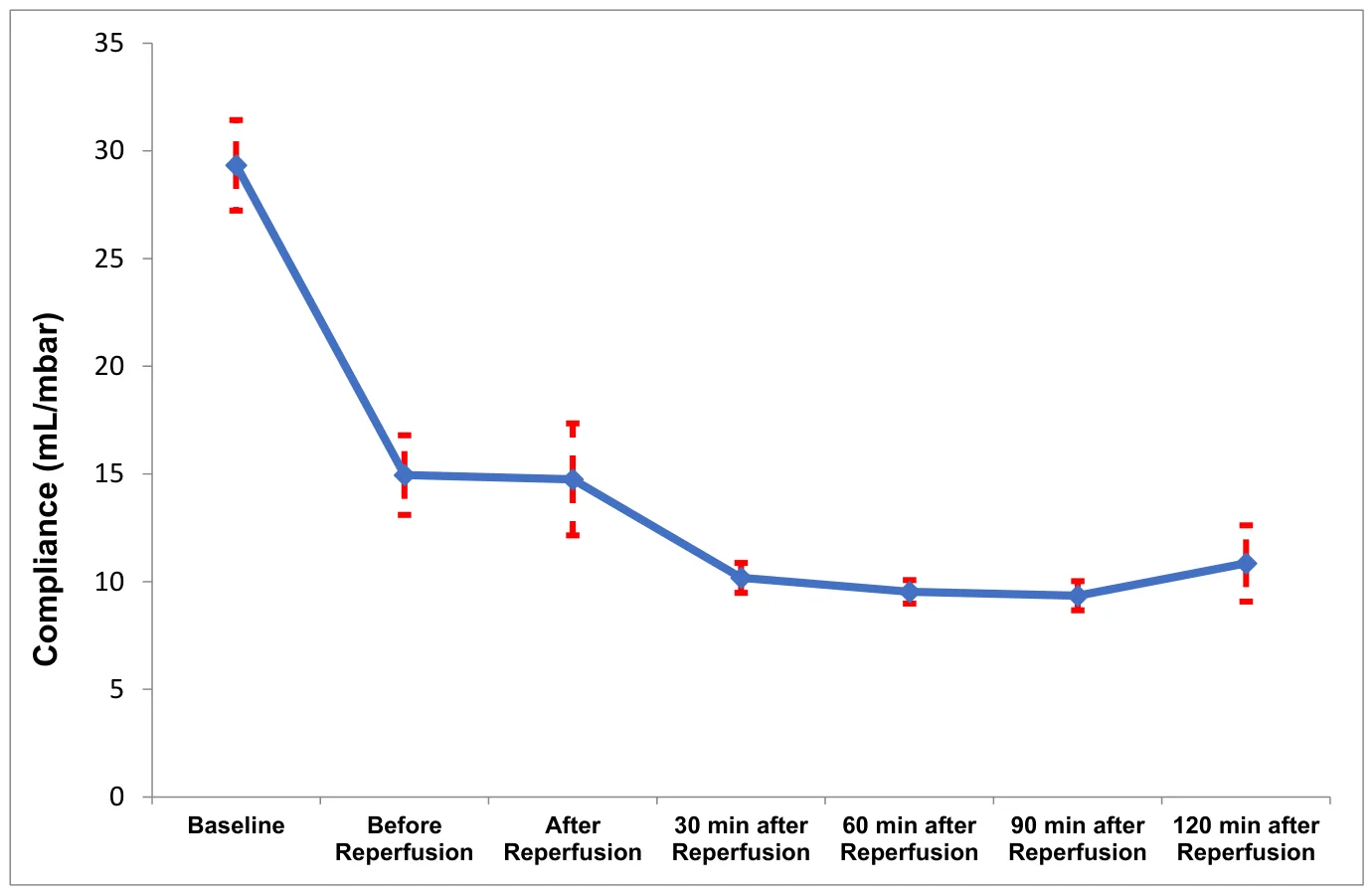
Changes in lung compliance in a porcine model of IRI-induced ARDS. *Baseline: sample 0; before reperfusion: sample 1; after reperfusion: sample 2; 30 min after reperfusion: sample 3a; 60 min after reperfusion: sample 3b; 90 min after reperfusion: sample 3c; 120 min after reperfusion: sample 3d*.

Lung compliance declined markedly following ipsilateral lung clamping (*p* < 0.001), decreasing by approximately 50% compared with baseline (Figure 4). Compliance remained low immediately after reperfusion (~15 mL/mmHg) and declined further during the recovery phase, stabilizing at 10–11 mL/mmHg.

### 3.3. Leukocyte Response

**Figure 5:**
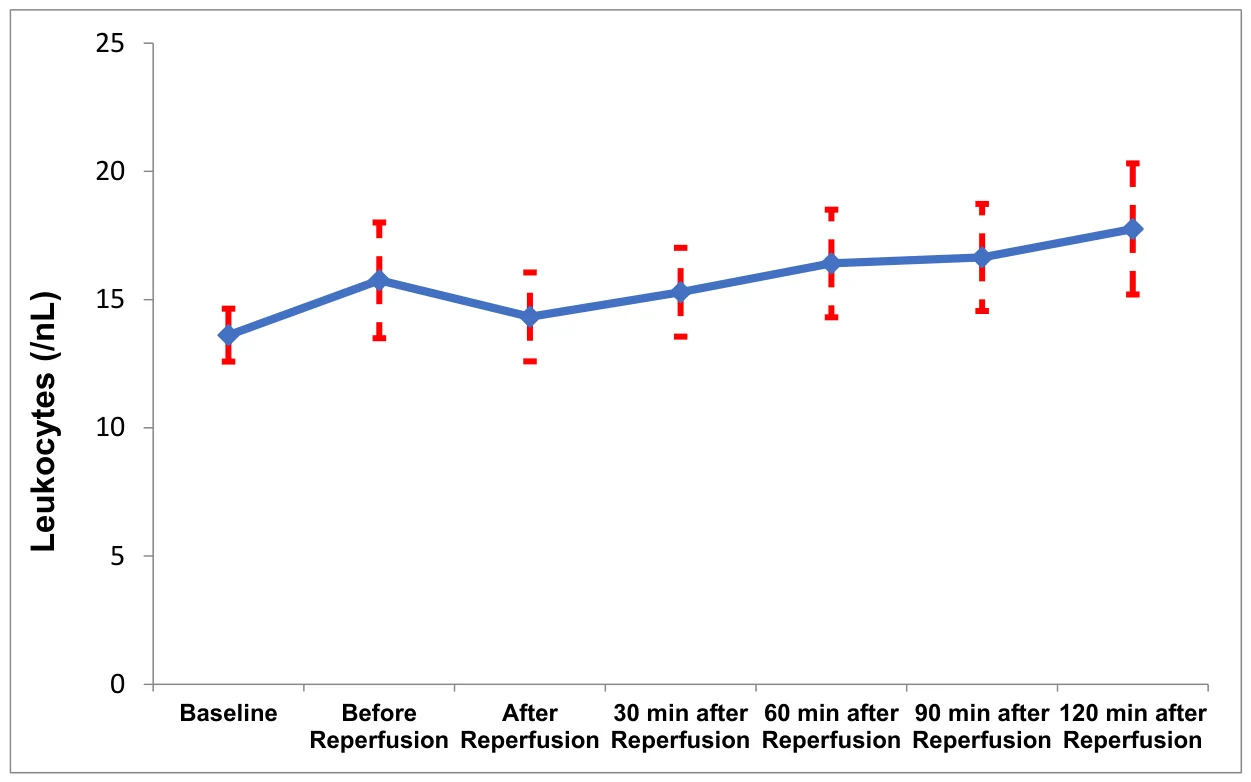
Changes in leukocyte counts in a porcine model of IRI-induced ARDS. *Baseline: sample 0; before reperfusion: sample 1; after reperfusion: sample 2; 30 min after reperfusion: sample 3a; 60 min after reperfusion: sample 3b; 90 min after reperfusion: sample 3c; 120 min after reperfusion: sample 3d*.

Leukocyte counts did not increase significantly during ARDS induction (*p* = 0.130) and continued to rise steadily throughout the experiment (Figure 5). Mean leukocyte values increased from 8.9 /nL at baseline to 21.3 /nL at 120 min after reperfusion.

### 3.4. Cytokine Levels (TNF-α and IL-6)

**Figure 6:**
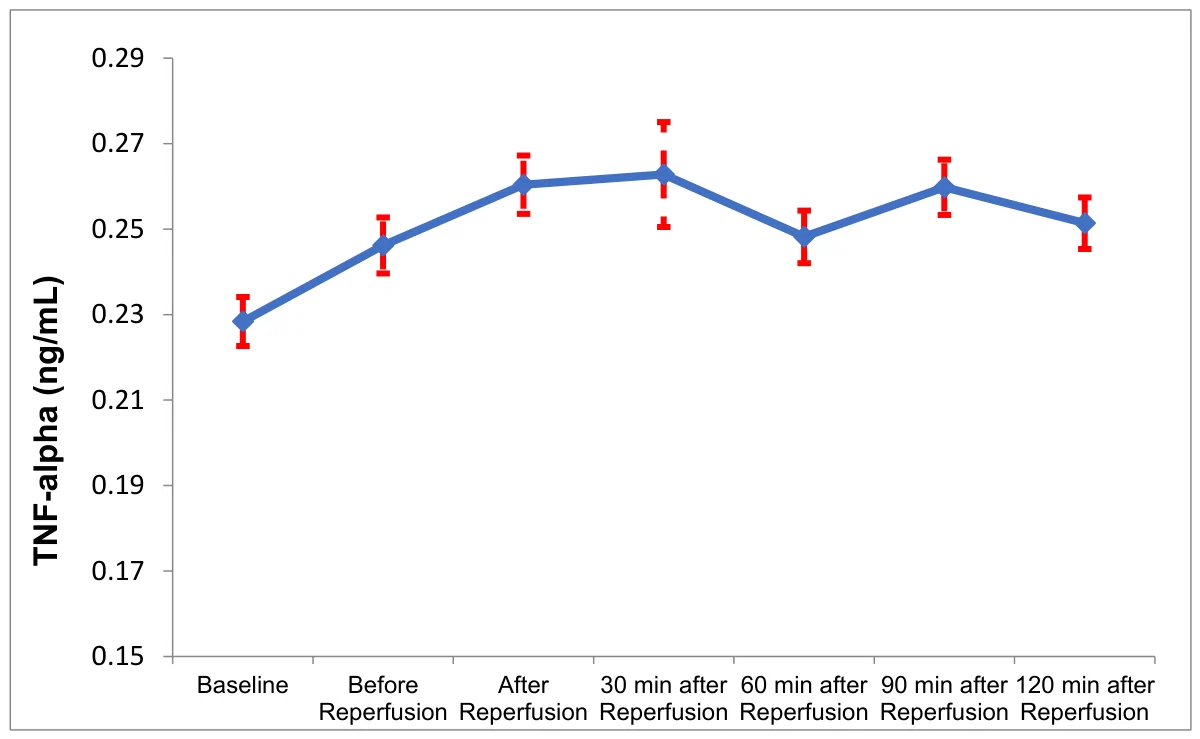
Changes in TNF-α concentrations in a porcine model of IRI-induced ARDS. *Baseline: sample 0; before reperfusion: sample 1; after reperfusion: sample 2; 30 min after reperfusion: sample 3a; 60 min after reperfusion: sample 3b; 90 min after reperfusion: sample 3c; 120 min after reperfusion: sample 3d*.

Serum TNF-α concentrations increased significantly during pulmonary ischemia–reperfusion injury compared with baseline values (*p* = 0.035). TNF-α levels increased rapidly after reperfusion and stabilized at approximately 0.25 ng/mL during the later observation period, indicating early activation of the pro-inflammatory signaling pathway associated with acute lung injury (Figure 6).

**Figure 7:**
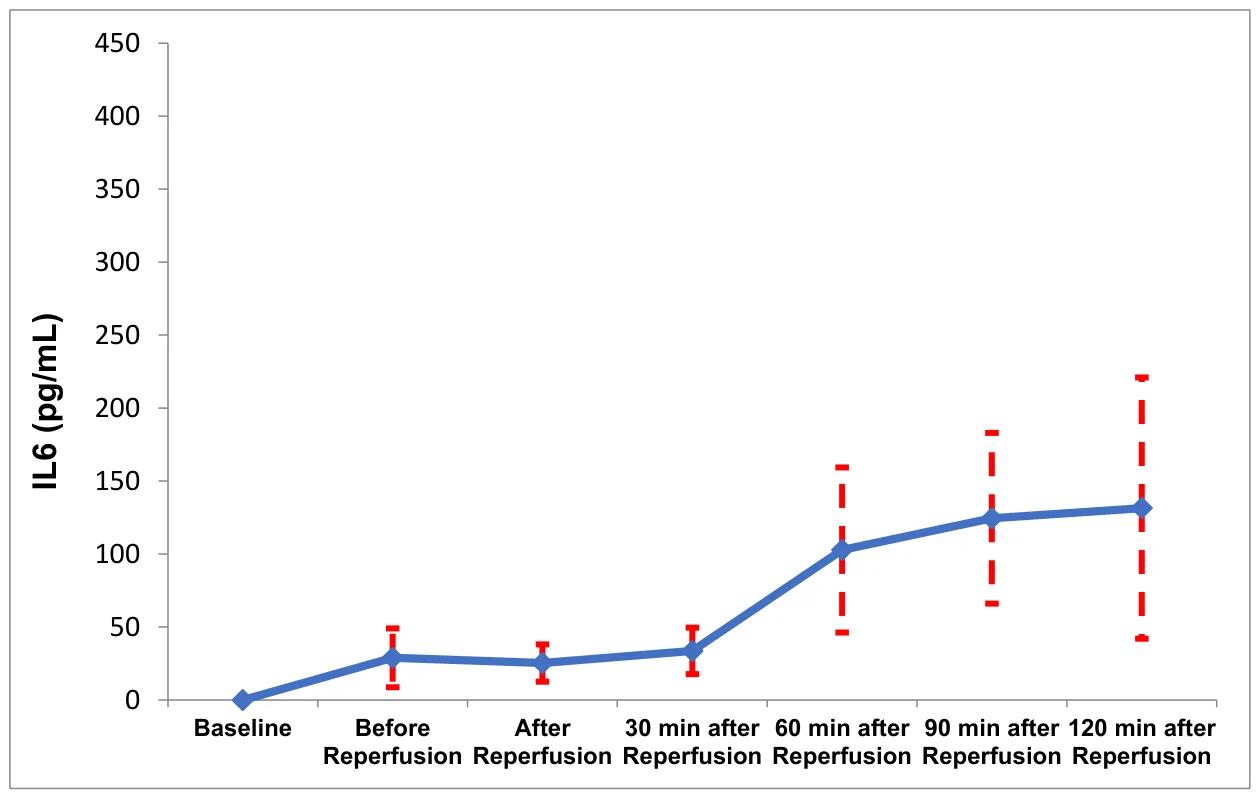
Changes in IL-6 concentrations in a porcine model of IRI-induced ARDS. *Baseline: sample 0; before reperfusion: sample 1; after reperfusion: sample 2; 30 min after reperfusion: sample 3a; 60 min after reperfusion: sample 3b; 90 min after reperfusion: sample 3c; 120 min after reperfusion: sample 3d*.

Baseline IL-6 concentrations were at or close to the lower detection limit of the assay. Following reperfusion, IL-6 concentrations increased markedly and continued to rise progressively throughout the observation period (*p* = 0.001), reaching peak concentrations 120 min after reperfusion (Figure 7). This sustained increase reflects the pronounced systemic inflammatory response associated with pulmonary ischemia–reperfusion injury and consistent with the early inflammatory phase of ARDS.

### 3.5. Histopathological Confirmation of ARDS and Neutrophil Infiltration

**Figure 8:**
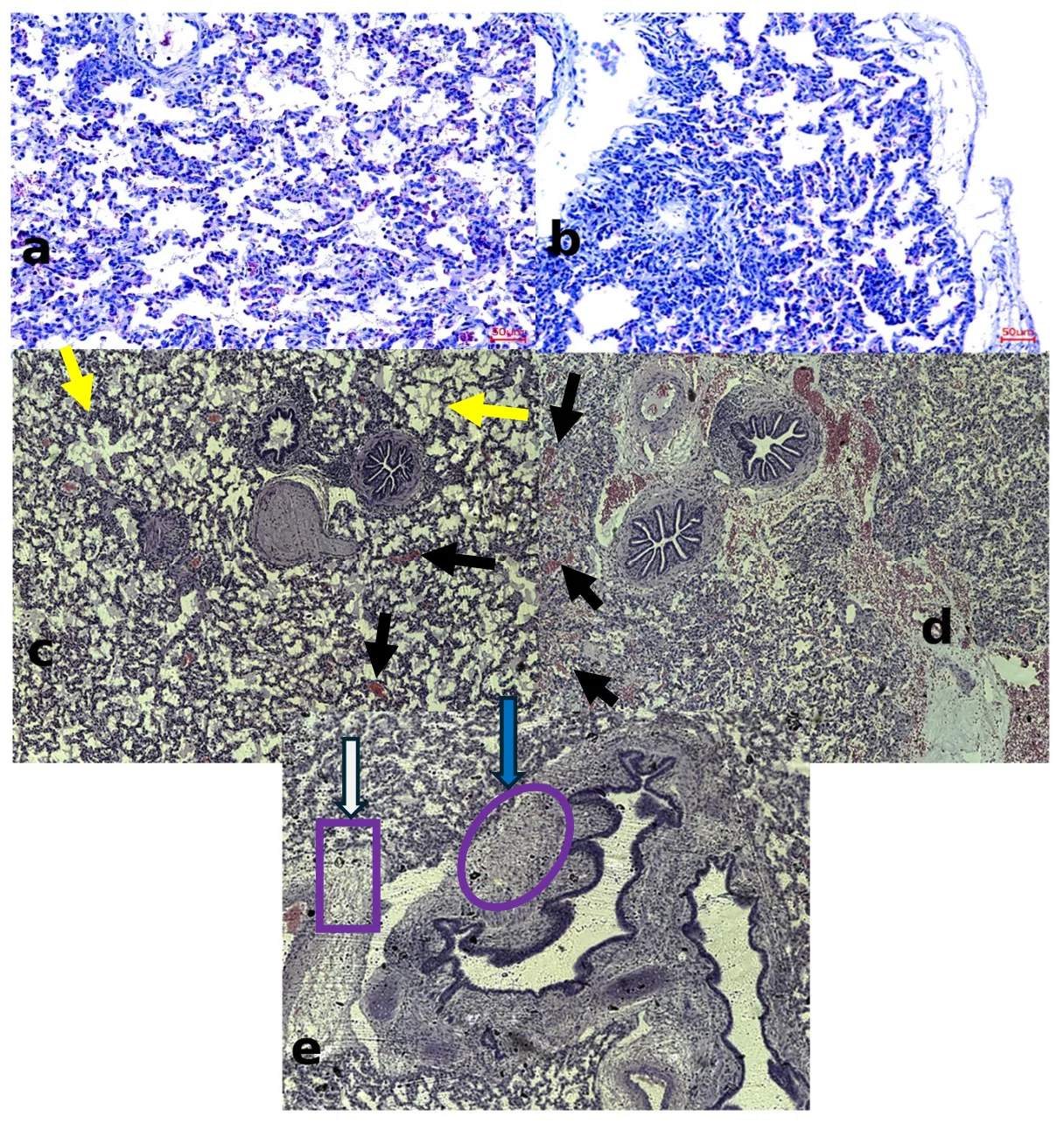
Representative histological sections obtained at baseline lung biopsies (**a**,**b**) and lung biopsies demonstrating characteristic features of early ARDS following pulmonary ischemia–reperfusion injury (**c**–**e**). Black arrows indicate intra-alveolar hemorrhage, yellow arrows indicate alveolar damage, the white arrow indicates perivascular edema, and the blue arrow indicates peribronchial edema.

Baseline lung biopsies demonstrated preserved pulmonary architecture with no evidence of interstitial edema, intra-alveolar hemorrhage, or inflammatory tissue infiltration. Small numbers of neutrophils were observed predominantly within the pulmonary capillary and interstitial compartments, consistent with the physiological presence of pulmonary neutrophils. The baseline biopsy was obtained after induction of general anesthesia, mechanical ventilation, thoracotomy, hilar preparation, and the predefined stabilization period, but before induction of pulmonary ischemia. Therefore, mild perioperative neutrophil sequestration associated with these preparatory procedures cannot be excluded. Importantly, these specimens showed no histopathological evidence of ARDS (Figure 8a,b).

**Figure 9:**
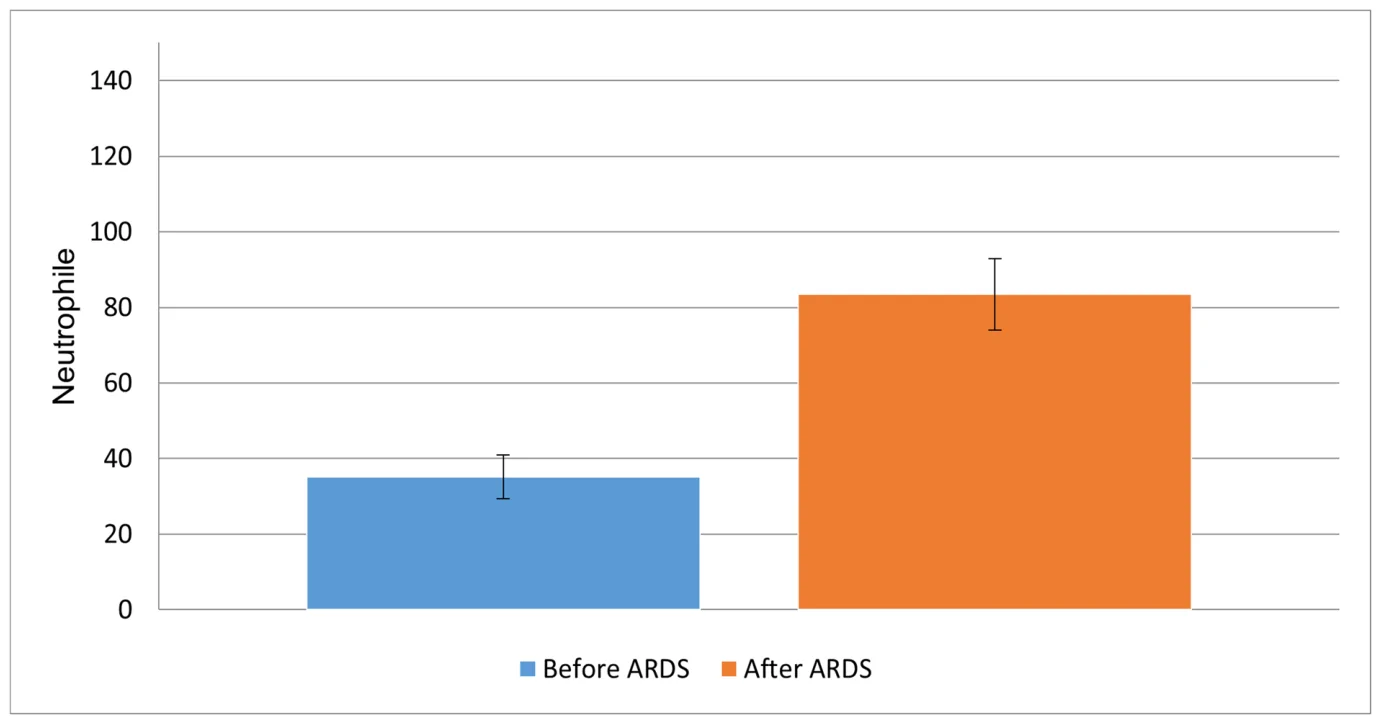
Mean neutrophil count in lung biopsies collected at baseline and at 120 min after reperfusion in a porcine model of IRI-induced ARDS.

All biopsies collected after reperfusion showed hallmark features of initial ARDS, including perivascular and interstitial edema as well as intra-alveolar hemorrhage (Figure 8c–e). All 10 post-reperfusion samples fulfilled all three histopathological criteria of ARDS. Quantitative assessment demonstrated a significant increase in neutrophil infiltration in ARDS specimens compared with control lung tissue (84.7 ± 14.3 vs. 35.2 ± 5.8 cells/HPF, paired Student’s *t*-test, *p* = 0.023, Figure 9), confirming a marked increase in pulmonary inflammatory cell infiltration following ARDS induction.

## 4. Discussion

Postoperative pulmonary complications after esophagectomy, especially acute respiratory distress syndrome (ARDS), remain the leading cause of mortality, accounting for up to 60% of in-hospital deaths [5,11,12]. ARDS occurs in about one-third of patients after transthoracic esophagectomy, with mortality rates exceeding 50% [5,8,10,12]. Pulmonary IRI is considered one of the principal mechanisms contributing to postoperative ARDS, which has not been well-represented in previous experimental models. Models like surfactant lavage or oleic acid administration do not reflect the pathophysiology of thoracic surgery [9]. In this study, we aimed to establish a clinically relevant large-animal ARDS model that mimics IRI-induced lung injury after esophagectomy, providing a platform to study the underlying mechanisms of IRI-induced ARDS and test preventive or therapeutic strategies for ARDS.

Although the present model does not reproduce the complete surgical procedure of esophagectomy, it was intentionally designed to isolate pulmonary ischemia–reperfusion injury, one of the major mechanisms implicated in postoperative ARDS. This approach allows controlled investigation of ischemia–reperfusion-related lung injury while minimizing confounding effects introduced by additional surgical and perioperative variables. We acknowledge that complete hilar inflow occlusion for several hours represents a standardized experimental insult that is likely more severe than the degree of pulmonary ischemia encountered during routine esophagectomy. However, this approach was intentionally chosen to achieve reproducible pulmonary ischemia–reperfusion injury of sufficient magnitude to permit consistent physiological, inflammatory, and histopathological characterization across animals. Nevertheless, this experimental approach cannot fully reproduce the complexity of clinical post-esophagectomy ARDS. The absence of esophagectomy, one-lung ventilation, perioperative fluid shifts, surgical manipulation, and postoperative systemic responses limits direct extrapolation to the clinical setting. Therefore, the model should be regarded as reproducing pulmonary ischemia–reperfusion injury relevant to postoperative ARDS rather than the complete postoperative syndrome itself.

### 4.1. Selection of the Large Animal Model

We chose to develop a large-animal model rather than a small-animal model to provide an essential bridge between experimental research and clinical application. Although large-animal models are more complex and resource-intensive than small-animal models, they are more relevant to the clinical situation because they are anatomically and physiologically more similar to humans, particularly with regard to lung architecture, respiratory monitoring, immune responses and cardiopulmonary interactions. Moreover, they allow comprehensive assessment of hemodynamic changes and physiological responses under experimental conditions [17]. Furthermore, large-animal models have already been used to improve ARDS management and to evaluate preventive strategies [14,15]; therefore, our study can build on these existing data. In this study, we developed a porcine model because of the aforementioned similarities and because pigs are suitable for prolonged experimental protocols with detailed assessment of gas exchange, lung perfusion, and systemic and pulmonary hemodynamics [14].

### 4.2. Induction of ARDS by Direct Pulmonary IRI

ARDS can be induced in experimental models via direct or indirect pathways. In this study, ARDS was induced by IRI, which represents a direct form of lung injury. This is often seen in clinical settings like thoracic surgery, lung contusions, and pneumonia [11,18]. This model reproduces endothelial injury in the pulmonary capillary bed after ischemia and reperfusion. In our model, we induced right-sided lung ischemia because it mirrors the clinical situation during transthoracic esophagectomy and maximizes the ischemic lung volume [5]. Furthermore, we included both ischemia and reperfusion phases because both are key factors in the severity of lung injury.

Lung oxygenation comes from the pulmonary artery, bronchial artery, and alveolar ventilation, but the specific contributions during pulmonary IRI are not well understood. Ischemia can occur with ventilated lungs (where blood flow is blocked but ventilation continues) or anoxic ischemia (where both blood flow and ventilation are blocked) [4]. Blood flow interruption in animal models can be achieved through clamping, ligation, or balloon occlusion [8], and each method results in different lung damage patterns [18,19]. We induced lung ischemia by clamping the hilar inflow of the lungs. Complete vascular clamping causes severe damage, including edema, hemorrhage, and inflammation [16]. Ventilation during ischemia worsens lung injury, likely due to increased vascular permeability and oxidative stress [10,18]. Oxygen exposure during ischemia generates reactive oxygen species, with injury severity correlating to ischemia duration [11]. This damage worsens with ischemia times longer than 60 min [4,20], so we opted to continue the experiment for 120 min to fully monitor the effects of the damage. Reperfusion, the most damaging phase, triggers inflammatory responses, leukocyte activation, and impaired ventilation–perfusion matching [21]. Reperfusion injury has two phases: an early neutrophil-independent phase within 30 min, followed by a delayed neutrophil-dependent phase [11]. Histopathological changes such as intra-alveolar edema, hemorrhage, and fibrin deposition occur early after reperfusion, accompanied by rapid increases in proinflammatory cytokines such as IL-6, which peak within hours after reperfusion and subsequently decline [20].

### 4.3. Hemodynamic Stability and Acid–Base Disturbances

We wanted to determine whether our experimental model is hemodynamically stable and reproducible because this is important for the validity of the model. In our model, cardiovascular parameters changed during ischemia and reperfusion, with predictable decreases in MAP and CVP and compensatory increase in heart rate. Despite these transient physiological alterations, cardiovascular stability was maintained, confirming the robustness and reproducibility of the model.

We also examined the changes in arterial pH in our model. Unilateral lung ischemia and reperfusion led to a significant and progressive decrease in arterial pH, reflecting systemic metabolic acidosis associated with pulmonary IRI. Concurrent respiratory acidosis was effectively counteracted by ventilatory adjustments. The subsequent return to normal pH during reperfusion in pulmonary IRI can induce cell death and subsequent organ dysfunction and gas exchange imbalance [22]. In line with this, and consistent with previous findings, we observed more pronounced lung injury during reperfusion than during ischemia alone [4]. These findings confirm that pH monitoring reliably captured systemic metabolic disturbances during ARDS induction.

### 4.4. Oxygenation Impairment and Lung Compliance

We assessed the efficiency of lung oxygenation by measuring the PaO_2_/FiO_2_ ratio, which showed a rapid, sustained and significant decline in oxygenation after reperfusion, falling below 200 mmHg within 30 min and remaining below 150 mmHg throughout the observation period. This oxygenation impairment corresponds to the physiological oxygenation criterion of moderate ARDS according to the Berlin Definition and was supported by histopathological findings consistent with acute lung injury [16]. Oxygenation remained severely impaired throughout the entire observation period, confirming that our ARDS model is both robust and effective. We also assessed lung compliance in our model, which decreased by approximately 50% after ipsilateral ischemia and declined even further after reperfusion. The initial decline in lung compliance reflected the exclusion of one lung from ventilation, whereas the secondary decrease after reperfusion indicated structural lung injury, increased alveolar permeability, and interstitial edema caused by IRI-induced ARDS. These findings reflect reported hallmark features of early ARDS such as increased pulmonary resistance, increased alveolar-capillary permeability, surfactant dysfunction, and interstitial edema [13,23,24]. In agreement with our findings, both experimental and clinical studies have shown strong inverse relationships between lung compliance and ARDS severity [5,12,13].

### 4.5. Inflammatory Cell Response and Proinflammatory Cytokine Activation

Consistent with IRI-induced inflammation, leukocyte counts did not increase significantly in our model during ARDS induction. The initial rise in leukocyte counts may reflect the effect of surgical stress and anesthesia, whereas the sustained increase observed during the later phases may be related to a systemic inflammatory response triggered by pulmonary IRI. However, because a sham-operated control group was not included, these potential contributing factors cannot be distinguished definitively in the present study. Nevertheless, pulmonary IRI is known to activate systemic and local inflammatory pathways [12]. For example, neutrophil activation plays a central role in post-IRI lung injury, leading to the production of reactive oxygen species and reactive nitrogen intermediates, which damage both alveolar epithelium and pulmonary endothelium [10,11,20]. The resulting increase in vascular permeability and release of inflammatory mediators results in pulmonary edema, pneumocyte apoptosis, and progression to ARDS [4,10,25].

We observed a marked increase in concentrations of the proinflammatory cytokines TNF-α and IL-6 during ischemia and early reperfusion. This is consistent with their established role as central mediators of the inflammatory response in ARDS pathogenesis. Specifically, TNF-α and IL-6 promote endothelial activation, neutrophil recruitment, and vascular permeability, which amplifies the inflammatory response. Notably, IL-6 exhibited a markedly stronger increase than TNF-α in our model, highlighting its value as a sensitive early biomarker for ARDS development [25,26].

### 4.6. Histopathological Validation of the ARDS Model

We also validated the histopathological characteristics of ARDS in our model. All lung biopsies collected from the animals after reperfusion showed classic features of the initial phase of ARDS, including congestion, interstitial edema, intra-alveolar hemorrhage, and neutrophil infiltration [27,28,29]. Because of the acute nature of our model, we only collected biopsies during the early phase of ARDS, when diffuse alveolar damage predominates. Neutrophil counts doubled within 2 h of ARDS induction, confirming early inflammatory lung injury and validating our physiological and biochemical findings [30], and corroborating that our model reliably reproduces the characteristics of early ARDS pathology in humans.

Despite the encouraging findings, several limitations of the present study should be acknowledged. First, even though the model was meant to recreate pulmonary ischemia–reperfusion injury during transthoracic esophageal surgery, it did not include a full esophagectomy. So, it misses the complete physiological stress associated with the clinical procedure. Second, there was not a sham-operated control group, which makes it tough to separate the effects of thoracotomy and mechanical ventilation from the actual ischemia–reperfusion injury itself. Third, the observation period was limited to two hours after reperfusion, capturing only the early phase of ARDS rather than its later stages. In addition, the sample size was relatively small, which is common in large-animal studies. However, it may limit the generalizability of the findings. Finally, the experimental protocol used a relatively severe and standardized period of unilateral pulmonary ischemia to make sure the lung injury showed up reliably. Although this approach resulted in highly reproducible ARDS features, the severity and duration of ischemia may not reflect the full spectrum of ischemic insults encountered during routine clinical esophagectomy. Looking ahead, there is a need for studies that test different durations and reperfusion protocols to determine the minimum injury threshold required to reliably induce clinically relevant ARDS while more closely reflecting real-world surgical conditions. Accordingly, the present model should be regarded as a translational platform for investigating pulmonary ischemia–reperfusion injury rather than as a complete experimental representation of post-esophagectomy ARDS.

In conclusion, we established a reproducible porcine model of pulmonary ischemia–reperfusion injury that reproduces key physiological, inflammatory, and histopathological features consistent with ARDS-like lung injury. The model demonstrated impaired oxygenation, reduced pulmonary compliance, systemic inflammatory activation, and characteristic histopathological changes consistent with early pulmonary ischemia–reperfusion-induced lung injury. Although it does not reproduce the complete clinical syndrome of post-esophagectomy ARDS, it provides a reproducible translational platform for investigating pulmonary ischemia–reperfusion injury and evaluating potential therapeutic strategies relevant to postoperative ARDS.

## Figures and Tables

**Table 1:** Summary of cardiocirculatory parameters in a porcine model of IRI-induced ARDS.

	Onset	Baseline	Before Reperfusion	After Reperfusion	30 min After Reperfusion	60 min After Reperfusion	90 min After Reperfusion	120 min After Reperfusion
Values								
**Pulse (/min)**		83.6 ± 8	71.5 ± 29.5	79.5 ± 33.3	100.3 ± 34.8	89.85 ± 35.5	112.5 ± 39.1	115 ± 39.6
**MAP (mmHg)**		62.1 ± 6.1	45.2 ± 19.4	42.8 ± 23	79.5 ± 23.1	45.5 ± 17.9	42.8 ± 16.6	40.4 ± 15.4
**CVP (mmHg)**		7.8 ± 1.0	9.5 ± 3.1	7.0 ± 1.9	6.1 ± 1.7	6.1 ± 1.6	5.5 ± 1.2	5.1 ± 1.0

Data are presented as mean ± standard deviation; *n* = 10 Landrace pigs. **Abbreviations:** ARDS = acute respiratory distress syndrome, CVP = central venous pressure, IRI = ischemia–reperfusion injury, MAP = mean arterial pressure. *Respiratory parameters (pH, PaO_2_/FiO_2_ ratio, lung compliance). Baseline: sample 0; before reperfusion: sample 1; after reperfusion: sample 2; 30 min after reperfusion: sample 3a; 60 min after reperfusion: sample 3b; 90 min after reperfusion: sample 3c; 120 min after reperfusion: sample 3d*.

## Data Availability

The data supporting the findings of this study are available from the corresponding author and approval by the Ethics Committee of Heidelberg University upon reasonable request.

## Supplementary Materials

The following supporting information can be downloaded at: https://www.mdpi.com/article/10.3390/medsci14040490/s1, Supplementary file: ARRIVE Checklist.

## Abbreviations

The following abbreviations are used in this manuscript:

ARDS	Acute Respiratory Distress Syndrome
MAP	Mean arterial pressure
CVP	Central vein pressure
IL-6	Interleukin-6
IRI	Ischemia–Reperfusion Injury
PIRI	Pulmonary Ischemia–Reperfusion Injury
ALI	Acute Lung Injury
PEEP	Positive End Expiratory Pressure
